# The Temporal Mechanisms Guiding Interneuron Differentiation in the Spinal Cord

**DOI:** 10.3390/ijms22158025

**Published:** 2021-07-27

**Authors:** Dylan Deska-Gauthier, Ying Zhang

**Affiliations:** Brain Repair Center, Department of Neuroscience, Faculty of Medicine, Dalhousie University, Halifax, NS B3H 4R2, Canada; Dylan.Deska-Gauthier@dal.ca

**Keywords:** spinal cord, interneuron, subpopulations, neurogenesis, mouse, zebra fish, temporal control, postmitotic differentiation, locomotion, sensory-motor control

## Abstract

Neurogenesis timing is an essential developmental mechanism for neuronal diversity and organization throughout the central nervous system. In the mouse spinal cord, growing evidence is beginning to reveal that neurogenesis timing acts in tandem with spatial molecular controls to diversify molecularly and functionally distinct post-mitotic interneuron subpopulations. Particularly, in some cases, this temporal ordering of interneuron differentiation has been shown to instruct specific sensorimotor circuit wirings. In zebrafish, in vivo preparations have revealed that sequential neurogenesis waves of interneurons and motor neurons form speed-dependent locomotor circuits throughout the spinal cord and brainstem. In the present review, we discuss temporal principals of interneuron diversity taken from both mouse and zebrafish systems highlighting how each can lend illuminating insights to the other. Moving forward, it is important to combine the collective knowledge from different systems to eventually understand how temporally regulated subpopulation function differentially across speed- and/or state-dependent sensorimotor movement tasks.

## 1. Introduction

Interneuron (IN) circuits in the spinal cord are essential for patterned, rhythmic and flexible motor control. From basic to complex sensorimotor tasks, combinatorial IN recruitments in the spinal cord are required for successful execution of movement. The spinal cord is comprised of vastly heterogeneous IN populations defined by unique molecular identities, intrinsic properties, connectivity and functional outputs. This IN diversity enables the spinal cord to coordinate varied movement schemes through dynamic environments. Thus, understanding spinal IN diversity and the developmental mechanisms that give rise to it, is fundamental to understanding movement.

Early work in the mammalian spinal cord revealed a remarkable spatial organization of progenitor domains along the dorsoventral axis during early embryogenesis [1,2]. These 11 progenitor domains give rise to distinct post-mitotic interneuron (IN) and motor neuron (MN) cardinal classes (dI1–dI6 INs, dILA–B, V0–V3 INs, MNs) defined by respective transcription factor (TF) expression profiles. Physiological and anatomical studies have revealed general connectivity, electrophysiological properties and functional outputs of these cardinal IN classes across various model systems [3,4,5]. However, extensive subpopulation heterogeneity has become evident within each cardinal class [6,7,8]. Furthermore, the developmental mechanisms underlying such subpopulation diversities are beginning to be understood.

Neurogenesis timing is an essential developmental mechanism for neuronal diversity and organization throughout the central nervous system [9,10]. Likewise, it plays an instructive role in the development of IN circuits within the spinal cord [11]. Notably, spinal INs form the final circuits controlling the coordination and rhythmicity of movement. This enables behavioural quantifications of their circuit outputs. Thus, spinal IN circuits are ideal model systems for understanding how differential neurogenesis timing contributes to molecular, cellular and behavioural development in the central nervous system.

To date, neurogenesis timing has been linked to post-mitotic molecular expression profiles, intrinsic membrane properties, circuit connectivities and behaviour-specific recruitments throughout IN populations in the spinal cord. In the present review, we focus on mouse and zebrafish model systems to explore how temporal controls of differentiation contribute to spinal IN diversity and corresponding behavioural flexibility.

## 2. Lessons from the Mouse Spinal Cord

### 2.1. Early Temporal Mechanisms Guide Molecular Diversity in the Mouse Spinal Cord

One of most revolutionary breakthroughs in understanding the development of spinal neurons is the discovery of spatially organized progenitors during early embryogenesis. Graded morphogens sonic hedgehog (Shh), released from the floor plate, and bone morphogenic protein (BMP)/Wnt protein, released from the roof plate, pattern the positions and cross-inhibitory boundaries of 11 discrete progenitor domains along the dorsoventral spinal axis [1]. These progenitor domains, in turn, give rise to 13 distinct post-mitotic cardinal IN populations and MNs. However, accumulating evidence suggests vast subpopulation diversity within each cardinal population and differential neurogenesis timing as a potentially key developmental mechanism for such diversity.

Recent work by Delile and colleagues utilized single cell RNA sequencing to systematically profile post-mitotic neurons across early embryonic stages (E9.5–E13.5) in the mouse spinal cord. They revealed a temporal emergence of shared transcription factor networks endowing subpopulation cluster identities across cardinal IN classes. This work was the first to systematically reveal previously underappreciated temporal mechanisms—acting in tandem with spatial controls—delineating IN subpopulation identities in the spinal cord. Indeed, several of these temporally regulated postmitotic TFs have been independently shown to be necessary for the specification and differentiation of subpopulation identities. Onecut TFs expressed across early-born spinal IN classes [12] are necessary for the differentiation of Renshaw cells (RCs) [13] and other spinal INs [14,15]; Pou2f2 and Zfhx TFs expressed across intermediate-born spinal IN classes [12] are necessary for proper migration [14,16] and molecular specification of laterally positioned V2a INs [17]; lastly, Nfib TFs expressed across late-born spinal IN classes [12] serve as molecular markers for a medial V2a IN subpopulation [17]. Together, this work has illuminated those neurons across the spinal cord may follow shared developmental temporal logic in their molecular diversification from spatially confined progenitor domains. However, understanding how and whether temporal mechanisms translate into distinct IN phenotypes, circuit integrations and functional outputs remains an ongoing question. Over the last decade, various studies have begun to investigate how differential neurogenesis timing orders the divergence of IN properties and functions.

### 2.2. Interneuron Subpopulations Emerge from Temporally Separated Progenitors

V1 INs, defined by engrailed-1 TF expression, arise from the p1 progenitor domain between embryonic days (E) 9.5 and E12.5 in the mouse spinal cord [5,13]. They project ipsilaterally and form inhibitory contacts onto both MNs and other IN classes in the ventral spinal cord [2]. In the mouse, V1 INs have been shown to be necessary for increased locomotor speed [7,18] and flexor–extensor alternation during walking [19,20]. Several classically characterized spinal IN types, such as RCs and inhibitory Ia-INs, were shown to be part of the V1 IN lineage [21]. They were among the first groups of subpopulations recognized within the cardinal populations. However, the vast heterogeneity of V1 INs was not fully revealed until the combinatorial expression of 19 distinct TFs was shown to delineate approximately 50 distinct V1 subsets throughout the lumbar and thoracic spinal cord [22,23,24].

More interestingly, in addition to revealing RC and Ia-IN V1 lineage, the same research groups showed that RCs and Ia-INs emerge from the p1 progenitor domain at different embryonic timepoints. They revealed that V1 INs could be organized into two general waves of neurogenesis: early (E9.5–E10.5) and late (E11.5–E12.5). The first wave of neurogenesis from the p1 progenitor domain gives rise to RCs [13,18], while the second wave gives rise to inhibitory Ia-INs, FoxP2^+^ V1 INs and other V1 IN subpopulations [18]. Early-born RCs are marked by the expression of a distinct TF profile (Foxd3, MafB, Onecut1 and Onecut2), as well as the calcium binding protein, calbindin [25,26]. Upon exiting from the p1 progenitor domain, RCs display a distinct ventrolateral migratory stream settling amongst lateral motor column MNs [25,27]. This early differentiation pathway allows RCs to form unique recurrent inhibitory circuits with MNs [26].

The early V1 IN birthdate determines a temporally ordered TF cascade necessary for the specification and maintenance of a RC-type specific phenotype [26]. Transcription factors Onecut1, Onecut2 and Foxd3 are responsible for the immediate postmitotic differentiation of RCs, including their calbindin expression, migration and circuit formation with MNs. Subsequently, downstream MafB expression is necessary for the maintenance of RC identity during late embryonic stages [26]. Thus, the specific early neurogenesis timing results in the postmitotic acquirement of a distinct TF expression cascade that facilitates the differentiation, maturation and circuit integration of RCs, separating them from other V1 INs.

While V1 IN subpopulation identity is correlated with neurogenesis time, to what extent their temporal expression profiles are endowed by intrinsic transcription programs or extrinsic signaling pathways remains an ongoing question. To begin to answer this question, Hoang and colleagues [28] established an in vitro model system of V1 IN diversification utilizing mouse embryonic stem cell (ESC) cultures. ESC V1 IN clades displayed transcription factor expressions, electrophysiological properties and connectivities that recapitulated those observed in the spinal cord [6,28]. Interestingly, ESC V1 IN subpopulations also displayed distinct neurogenesis birth orders in culture. Calbindin^+^ V1 INs were born first, followed by Foxp2^+^ V1 INs. These results, from a system in the absence of many surrounding extrinsic signaling sources, are similar to those observed in vivo [25,26].

Hoang and colleagues [28] further assessed a potential causative link between neurogenesis timing and subpopulation generation. Through inhibition of Notch signaling, they were able to increase the rate of cell cycle exit and neurogenesis timing. When Notch was inhibited at early stages, there was a significant increase in the proportion of early-born Calbindin^+^ V1 INs, as well as other TFs belonging to the MafA^+^ V1 clade. This early-born subpopulation increase was accompanied by an almost complete loss of late-born Foxp2^+^ V1 INs. These experiments indicated that, when late-born V1 INs were prematurely pushed out of the p1 progenitor domain, they switched to an early-born subpopulation fate.

Taken together, both these in vivo and in vitro studies suggest that differential neurogenesis timing enables specific temporal transcription pathways in p1 progenitor cells to be translated into distinct V1 IN subpopulation fates. That is, the transcriptional identity of a V1 IN (or any spinal IN) at the time it becomes post-mitotic may instruct its subpopulation fate choice.

Beyond V1 INs, we have begun to investigate how neurogenesis timing underlies subpopulation divergence within the most ventral-originating V3 IN cardinal class. V3 INs, marked by TF Sim1 expression, also exit from the p3 progenitor domain between E9.5 and E12.5 in the mouse spinal cord. V3 INs are mostly commissural and excitatory INs [29,30]. They are functionally involved in coordinating excitation between left–right extensor centres [31] and robust locomotor pattern output [30]. As V3 INs become postmitotic, they form distinct dorsolateral and ventrolateral migratory streams. Early-born (E9.5–E10.5) V3 INs follow both dorsolateral and ventrolateral migratory streams and cluster across deep dorsal, intermediate and ventral laminae by postnatal day (P) 0. In contrast, late-born (E11.5–E12.5) V3 INs almost exclusively follow the ventrolateral migratory stream and cluster mostly within ventral laminae by P0 [32]. Furthermore, early-born V3 INs display both ascending and descending axonal projections, while late born V3 INs display significantly more descending axonal projections than ascending ones [32]. Thus, successive neurogenesis timing fate-restricts late-born V3 INs to anatomically confined subpopulations.

Whether distinct transcriptional pathways are restricted to different temporal waves of V3 neurogenesis, as seen in V1 INs, remains largely unknown. Though, we have shown some evidence that the Sim1 TF, while expressed in all V3 INs, exclusively affects the laminar clustering and electrophysiological properties of early-born dorsal and intermediate V3 IN clusters, but not late-born ventral V3 IN clusters. Much more work is required to reveal the role temporal mechanisms play in regulating the molecular pathways underlying V3 IN subpopulation diversity and how that diversity is then translated into functionally distinct circuit integrations.

### 2.3. Select Dorsal IN Populations Emerge from Temporally Separated Progenitors

Select sensory-related dorsal IN populations have also been shown to emerge during specific neurogenesis windows. Dorsal horn progenitors separate along both spatial and temporal axes of control. Two dorsal progenitor lineages, dILA and dILB INs, emerge from Lbx1^+^ dorsal progenitors during specifically late neurogenesis stages. dILA coexpress Gbx1 with Pax2 and Lbx1 and are inhibitory, while dILB coexpress Lmx1b with Lbx1 and are excitatory [33,34,35]. Of particular note, Gbx1 is exclusively expressed and is necessary for the specification and differentiation of late-born dILA INs [26,36,37].

Loss of Gbx1 resulted in abnormal hindlimb gaits during locomotion, as well as sensory processing [36,37]. Gbx1 knockout (KO) mice displayed a duck-type gait characterized by hyper flexion during the swing phase and a decrease in average locomotor speed in open field tests. They also displayed reduced thermal pain sensitivity and increased slips during beam walking [37]. However, whether this was due to Gbx1 early expression in the floor plate, ISL1^+^ motor neurons, or dILA INs is not clear. However, Gbx1 KO mice displayed intact motor strength and no changes in the number of ISL1^+^ MNs, nor sensory innervations patterns [37]. Taken together, this suggests that temporally regulated expression of Gbx1 in late-born dorsal INs is necessary for the specification of dILA INs, which are involved in distinct aspects of sensorimotor control.

Sensory mediating cerebrospinal fluid contacting neurons were also shown to have a characteristically late neurogenesis timing in the mouse spinal cord. These neurons are born as late as E14–E16 from the oligodendrocite and p2 progenitor domains [38]. This neurogenesis window is well beyond the common neurogenesis window observed in the mouse spinal cord. Cerebrospinal fluid contacting neurons settle around the central canal and display a unique morphology with the extension of a dendrite into the central canal, unique mechanosensitive channel expression and distinct electrophysiological properties [38]. While their function has not been shown in mice, they are likely functionally distinct from other p2 originating IN types, V2a and V2b INs. Indeed, in the zebrafish cerebrospinal fluid contacting neurons have been shown responsible for sensing spinal bending and mediating postural control during locomotion [39,40,41].

### 2.4. Neurogenesis Timing Can Restrict in Specific Circuit Wirings

Timing of neuronal differentiation may play a role beyond defining molecular identity of spinal subpopulations, to guiding the formation of distinct circuit connectivity. While limited studies have been performed to date, there is evidence for IN neurogenesis timing and motor pool specific wiring.

**Figure 1:**
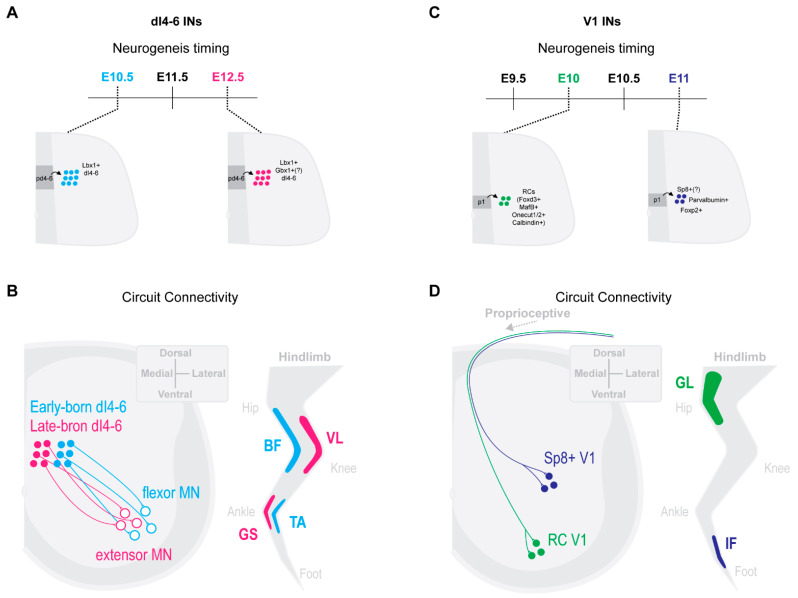
IN neurogenesis timing and circuit connectivity in the mouse spinal cord. (**A**) dI4-6 last-order INs emerge during either early-born (E10.5) or late-born (E12.5) neurogenesis waves [42]. Late-born dI4-6 INs are likely dILA/B IN populations and some likely express Gbx1^+^ [26,36,37]. (**B**) By postnatal stages, early-born dI4-6 INs are positioned preferentially laterally and innervate flexor MNs, while late-born dI4-6 INs are positioned preferentially medially and innervate extensor MNs [42] (extensor muscles, vastus lateralis (VL), gastrocnemius (GS); flexor muscles, biceps femoris (BF), Tibialis anterior (TA)). (**C**) Early-born V1 INs (E10) differentiate into Renshaw cells (RCs) [13,25], while late-born V1 INs (E11) (differentiate into Foxp2^+^ V1 INs [25], Parvalbumin+ V1 INs [25] and, likely, Sp8^+^ V1 INs [27]. (**D**) By postnatal stages, early-born RC V1 INs settle within more ventral clusters and receive more proprioceptive innervations from proximal hip muscles [6]. Presumptive later-born Sp8^+^ V1 INs [28] settle within more dorsal clusters and receive more proprioceptive innervations from distal foot muscles [6] (proximal muscle, Gluteus (GL); distal muscle, intrinsic foot (IF)).

Trans-synaptic viral tracing by Tripodi and colleges [42] revealed that ipsilateral dI4-6 INs, respectively, innervating flexor or extensor MNs, were spatially, synaptically and temporally separated. Last-order extensor INs were positioned more medially and received high levels of proprioceptive innervation, while flexor INs were positioned more laterally and received less proprioceptive innervations. Interestingly, last-order flexor INs were early-born cells (around E10.5), while last-order extensor INs were born later (around E12.5) (Figure 1A,B [42]). The late-born Lbx1^+^ last-order extensor INs were most likely from late dILA,B progenitors [24,30,38,43]. Thus, the time within which an IN becomes post-mitotic may position it in a specific functional pathway.

Likewise, neurogenetically separated V1 INs display distinct microcircuit connectivity. Early-born V1 RCs [13,25] settled within more ventral clusters and received more proprioceptive innervations from proximal hip muscles [6]. In contrast, presumptive later-born Sp8^+^ V1 INs [28] settled within more dorsal clusters and received more proprioceptive innervations from distal foot muscles [6] (Figure 1C,D). While the functional relevance of these distinct sensory innervation patterns remains to be determined, it suggests an intriguing link between timing of an INs differentiation and microcircuit specific integration.

## 3. Lessons from the Zebrafish: Sequential Waves of Neurogenesis form ‘Layered’ Locomotor Circuits in the Zebrafish Spinal Cord and Brainstem

### 3.1. Early Maturation of Swimming Behaviours Is Underscored by Sequential Waves of Neurogenesis

The zebrafish model has enabled a linking of neuronal lineages to their circuit connectivity and in vivo functional recruitments. Locomotor speed dependent circuits have been extensively studied in the zebrafish spinal cord [44]. Spinal MN and IN subtypes display varied speed-dependent recruitment properties enabling locomotor frequency regulation.

Neurogenesis timing is a key determinant in the formation of speed dependent circuits in the zebrafish spinal cord. Larval zebrafish sequentially develop distinct locomotor behaviours at set postfertilization (pf) timepoints. They first display exclusively large amplitude single tail bends around 1 day (d) pf, followed by high frequency and high amplitude burst swimming around 3 dpf and, finally, slow frequency, low amplitude and continuous swimming around 4–5 dpf [45,46,47,48,49,50]. This developmental timeline of locomotor flexibility is a readout of underlying developmental changes occurring early pf. Several developmental mechanisms occur during this time, including a maturation of neuronal intrinsic properties, a refining of synaptic connectivities (including a switch from electrical to chemical synapses) and the staggered neurogenesis of distinct spinal MN and IN types [44,45,46,47,48,49,50,51,52,53,54,55].

**Figure 2:**
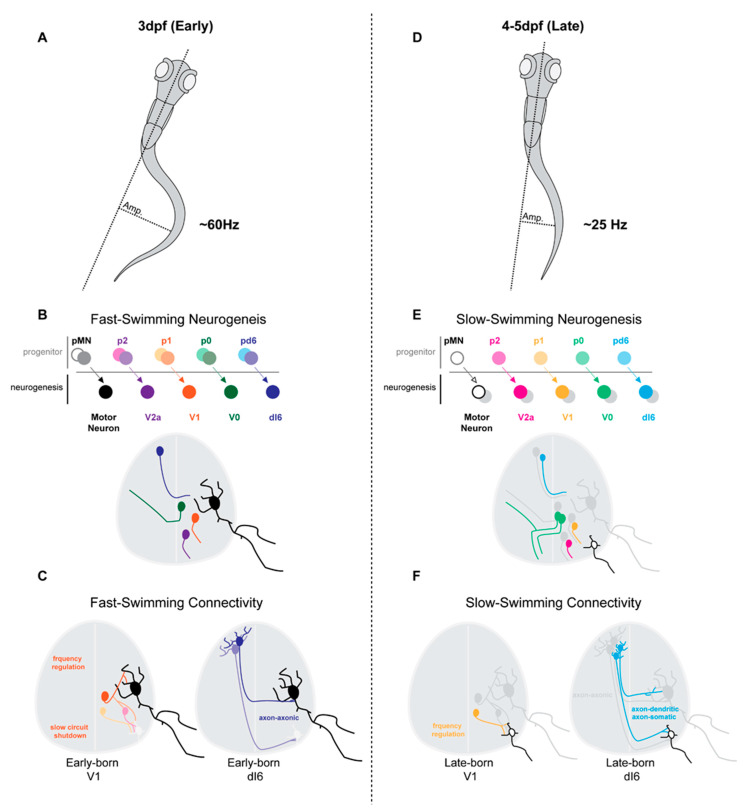
Sequential waves of neurogenesis separate zebrafish spinal cord neurons along neurogenesis time- and speed-matched axes. Large amplitude (Amp) and high frequency swimming emerge early, at 3 days post feralization (dpf), in the zebrafish larvae (**A**). Small amplitude (Amp) and low frequency swimming emerge later, at 4–5 days post feralization (dpf), in the zebrafish larvae (**B**) [48,49]. Early maturation of swimming behaviours is underscored by sequential waves of neurogenesis (**C**), early-born and fast swimming recruitment; (**D**), late-born and slow swimming recruitment; motor neurons (MNs), [49]; V0, [48,49,55]; V2a INs, [52]; V1 INs, [53]; dI6 INs, [62]. Neurogenesis time- and speed-matched spinal IN subclasses display distinct circuit connectivities. Early-born V1 INs regulate MN burst termination of primary MNs during fast-swimming as well as slow-swimming circuit shutdown of slow speed V2a INs and secondary MNs [54]. Early-born dI6 INs form axon-axonic synaptic connectivities with MNs and are recruited during fast-swimming (**E**). Late-born V1 INs regulate MN burst termination of secondary MNs during slow-swimming. Late-born dI6 INs form axon-somatic and axon-dendritic synaptic connectivities with MNs and are recruited during slow-swimming (**F**) [54].

It may not be surprising, then, that McLean and colleagues [48,49] showed that spinal circuits involved in different swimming speeds display a temporal ordering of neurogenesis and differentiation. Spinal INs and MNs involved in high-amplitude and fast swimming speeds emerge first during early larval development (Figure 2A,B). Subsequently, INs and MNs involved in lower amplitude and slow swimming speeds differentiate (Figure 2C,D). This temporal ordering of speed-related swimming circuits results in a topographic recruitment map across the dorsoventral axis in the larval zebrafish spinal cord [48,49]. As larval swimming speeds increase, so do the recruitments of increasingly ventral INs and MNs. Interestingly, while speed-dependent circuits maintain a modular recruitment logic in the adult, they no longer display a clear topographic organization [56,57,58]. Thus, topographic organization in the larvae may be more representative of the sequential differentiation from fast to slow swimming circuits than final neuronal positioning.

### 3.2. Spinal Neurons Separate along Neurogenesis Time- and Speed-Matched Axes

Spinal MNs can be categorized as either primary or secondary MNs in embryonic and larval zebrafish. Primary MNs are born during an early neurogenesis wave and are recruited during large amplitude and fast frequency escape and swimming movements. Secondary MNs are born during a later neurogenesis wave and are recruited during slow frequency swimming [59]. Furthermore, primary and secondary MNs display unique morphological and electrophysiological properties. Primary MNs have larger soma sizes, smaller input resistances, more extensive dendritic branching, larger axon diameters and more ventromedial axon projection pathways and settle within relatively more dorsal positions than secondary MNs [59,60,61]. Primary and secondary MNs also express distinct calcium channel types, resulting in distinct neurotransmitter release properties and downstream muscle fibre control [62]. Thus, neurogenesis timing appears to serve as an early organizing principle for the anatomical and intrinsic properties of zebrafish MNs, resulting in fast and slow swimming control.

Beyond MNs, spinal INs in the zebrafish display strong correlations between their neurogenesis timings and functional recruitment patterns. Distinct excitatory and inhibitory spinal IN lineages integrate within fast- and slow-swimming circuits dependent on their neurogenesis timing. Similar to MNs and across all studied IN lineages, early-born spinal INs differentiate into fast-swimming circuits, while later-born spinal INs differentiate into slow-swimming circuits. Beyond speed matched recruitment patterns, the temporal ordering of IN differentiation also appears to play a key role in the establishment of subtype-specific intrinsic properties and connectivities.

Anatomically and neurochemically distinct V0 INs are produced in a time-dependent manner from heterogeneous p0 progenitor cells in the zebrafish spinal cord [55]. Commissural and excitatory V0-e INs display a correlation between their neurogenesis timing and axon projection profiles. Ascending commissural V0-e INs are born first, followed by bifurcating commissural V0-e INs and, finally, descending commissural V0-e INs. In this case, neurogenesis timing seems to play a direct role in ordering V0 INs axon projection phenotypes. Accelerated neurogenesis timing by reduced notch signaling resulted in an increased number of early-born ascending V0-e INs [55]. Thus, neurogenesis timing orders V0e INs down distinct differentiation pathways, resulting in temporally ordered axon projection profiles.

Late-born descending V0-e INs can be further subdivided into unipolar (UCoD) and multipolar (MCoD) commissural descending INs. Thus, further subpopulation heterogeneity exists within the late-born neurogenesis window of V0-e INs. Although MCoD INs were specifically recruited during slower swimming speeds in the larvae zebrafish [48,49], no correlation between morphology and speed-dependent recruitment of V0-e INs was found in the adult spinal cord [62]. Therefore, it is possible that either early morphological distinctions in the larvae are lost in the adult, or that V0-e IN recruitment patterns change with maturation.

In addition to excitatory INs, both commissural and ipsilateral inhibitory INs display neurogenesis matched properties and circuit integrations. Inhibitory and ipsilaterally projecting V1 INs can be generally divided into either early-born or later-born groups. Early-born V1 INs are preferentially recruited during higher swimming frequencies, while later-born V1 INs during slower swim frequencies [53]. When all V1 INs are ablated, fish display reduced swimming frequencies due to increased cycle periods across both fast and slow speeds. Additionally, during fast swimming bouts, MNs and INs from slow swimming circuits exhibit reduced inhibition [53]. Thus, both early-born fast-type and later-born slow-type V1 INs are involved in cycle-burst termination, required for swimming frequency (Figure 2C,F). However, fast-type V1 INs are proposed to further inhibit slow V2a IN and MN circuits during specifically high-speed swimming (Figure 2C). Therefore, the neurogenesis timing of V1 INs corresponds to both speed-specific recruitments and functional outputs.

### 3.3. Neurogenesis and Differentiation Timing Matches Pre- and Post-Synaptic Targets

Recent work from McLean’s group has just revealed even further that neurogenesis timing can order the subcellular innervation patterns of last-order inhibitory INs. Inhibitory commissural dI6 INs necessary for left–right alteration form distinct microcircuit connectivities with commissural MNs, depending on their neurogenesis times [35]. Temporally, morphologically and synaptically distinct dI6 IN circuits are differentially recruited and function across increasing swimming speeds. Early-born dI6 INs synapse primarily on MN axons and are recruited during highest frequency swimming (Figure 2C). This axonal innervation is likely functionally necessary for quick MN termination, needed for high frequency left–right alteration. Late-born dI6 INs primarily synapse onto MN somas and dendrites and are recruited during slower swimming speeds (Figure 2F) [63]. Interestingly, these temporally regulated synaptic IN–MN innervation patterns appear to be determined by the available post-synaptic targets at the time of dI6 IN neurogenesis [60]. As late-born dI6 INs exit the cell cycle, MNs begin to extend elaborate dendritic arbors [54], thus providing temporally aligned targets (dendrites) for the late-born dI6 INs. Taken together, this work suggests that neurogenesis timing may organize neural circuit formation by temporally layering the alignment of pre- and post-synaptic targets.

### 3.4. Temporal Layering of Spinal Circuits Extends to the Brainstem

Moving beyond connections within the spinal cord, the question remains of whether the temporal ordering of spinal circuits extends to peripheral and supraspinal inputs entering the spinal cord. Indeed, recent work by Pujala and colleagues [64] revealed that temporal neurogenesis ordering from fast to slow locomotor control is extended to circuits in the brain stem. Early-born hindbrain V2a neurons are recruited during fast locomotor bursts, while later-born hindbrain V2a INs are recruited during slower swimming movements [64]. Interestingly, descending hindbrain V2a neurons display age- and function-matched connectivity patterns with networks in the spinal cord. Early-born hindbrain V2a neurons form connections with fast locomotor networks in the spinal cord, while later-born hindbrain V2a form connections with slow swimming spinal networks [64]. Thus, the temporal layering of spinal circuits involved in fast–slow locomotor control appears to extend beyond the spinal cord to hindbrain motor circuits.

## 4. Concluding Remarks

Neurogenesis timing has been shown as a key developmental mechanism in patterning neuronal circuits across the nervous system of varied species [9,10]. Indeed, temporal TF networks have been revealed and extensively studied throughout lower invertebrate species, such as Drosophila [26] and C. elegans [30]. In these cases, a neuron postmitotic identity and fate choice is largely dependent on its dynamic TF state at the time it exits the cell cycle.

While such specific temporal TFs have yet to be revealed within mammalian spinal cord progenitor cells, from the collection of works presented here, they likely exist and play crucial roles in diversifying spinal IN identities emerging from within and between spatially confined progenitors. Indeed, work on the mouse has revealed that some temporally regulated postmitotic TF expressions in specific IN subpopulations instruct distinct circuit connectivity [12,13,14,15,16]. However, to date, an understanding of how temporally regulated post-mitotic TFs translate into subpopulation specific functional roles has remained largely unexamined in the mouse spinal cord. Yet, in the zebrafish spinal cord, while the molecular logic remains much less understood, several studies have demonstrated how temporally regulated IN types differentially contribute to fast and slow swimming circuits. Thus, moving forward, work conducted in each model system can lend illuminating insights to the other. Particularly, with the recent discovery of temporally regulated post-mitotic TFs in the mouse spinal cord, it will be interestingly to understand whether differential neurogenesis timing plays a role in determining subpopulation specific expression of these TFs and to what extent temporally regulated subpopulation fates differentially function across speed- and/or state-dependent sensorimotor tasks.

## References

[1] Jessell T.M. (2000). Neuronal Specification in the Spinal Cord: Inductive Signals and Transcriptional Codes. Nat. Rev. Genet..

[2] Lu D.C., Eniu T., Alaynick W.A. (2015). Molecular and Cellular Development of Spinal Cord Locomotor Circuitry. Front. Mol. Neurosci..

[3] Deska-Gauthier D., Zhang Y. (2019). The Functional Diversity of Spinal Interneurons and Locomotor Control. Curr. Opin. Physiol..

[4] Goulding M. (2009). Circuits Controlling Vertebrate Locomotion: Moving in a New Direction. Nat. Rev. Neurosci..

[5] Kiehn O. (2016). Decoding the Organization of Spinal Circuits That Control Locomotion. Nat. Rev. Neurosci..

[6] Bikoff J.B., Gabitto M.I., Rivard A.F., Drobac E., Machado T., Miri A., Brenner-Morton S., Famojure E., Diaz C., Alvarez F.J. (2016). Spinal Inhibitory Interneuron Diversity Delineates Variant Motor Microcircuits. Cell.

[7] Gosgnach S., Bikoff J.B., Dougherty K.J., El Manira A., Lanuza G.M., Zhang Y. (2017). Delineating the Diversity of Spinal In-ter-neurons in Locomotor Circuits. J. Neurosci..

[8] Ziskind-Conhaim L., Hochman S. (2017). Diversity of Molecularly Defined Spinal Interneurons Engaged in Mammalian Locomotor Pattern Generation. J. Neurophysiol..

[9] Holguera I., Desplan C. (2018). Neuronal Specification in Space and Time. Sci..

[10] Oberst P., Agirman G., Jabaudon D. (2019). Principles of Progenitor Temporal Patterning in the Developing Invertebrate and Ver-tebrate Nervous System. Curr. Opin. Neurobiol..

[11] Sagner A., Briscoe J. (2019). Establishing Neuronal Diversity in the Spinal Cord: A Time and a Place. Development.

[12] Delile J., Rayon T., Melchionda M., Edwards A., Briscoe J., Sagner A. (2019). Single Cell Transcriptomics Reveals Spatial and Temporal Dynamics of Gene Expression in the Developing Mouse Spinal Cord. Development.

[13] Stam F.J., Hendricks T.J., Zhang J., Geiman E.J., Francius C., Labosky P., Clotman F., Goulding M. (2012). Renshaw Cell In-ter-neuron Specialization Is Controlled by a Temporally Restricted Transcription Factor Program. Development.

[14] Harris A., Masgutova G., Collin A., Toch M., Hidalgo-Figueroa M., Jacob B., Corcoran L.M., Francius C., Clotman F. (2019). Onecut Factors and Pou2f2 Regulate the Distribution of V2 Interneurons in the Mouse Developing Spinal Cord. Front. Cell. Neurosci..

[15] Kabayiza K.U., Masgutova G., Harris A., Rucchin V., Jacob B., Clotman F. (2017). The Onecut Transcription Factors Regulate Differentiation and Distribution of Dorsal Interneurons During Spinal Cord Development. Front. Mol. Neurosci..

[16] Masgutova G., Harris A., Jacob B., Corcoran L.M., Clotman F. (2019). Pou2f2 Regulates the Distribution of Dorsal Interneurons in the Mouse Developing Spinal Cord. Front. Mol. Neurosci..

[17] Hayashi M., Hinckley C.A., Driscoll S.P., Moore N.J., Levine A., Hilde K.L., Sharma K., Pfaff S.L. (2018). Graded Arrays of Spinal and Supraspinal V2a Interneuron Subtypes Underlie Forelimb and Hindlimb Motor Control. Neuron.

[18] Falgairolle M., O’Donovan M.J. (2019). V1 interneurons regulate the pattern and frequency of locomotor-like activity in the neonatal mouse spinal cord. PLoS Biol..

[19] Zhang J., Lanuza G.M., Britz O., Wang Z., Siembab V.C., Zhang Y., Velasquez T., Alvarez F.J., Frank E., Goulding M. (2014). V1 and v2b interneurons secure the alternating flexor-extensor motor activity mice require for limbed locomotion. Neuron.

[20] Britz O., Zhang J., Grossmann K.S., Dyck J., Kim J.C., Dymecki S., Gosgnach S., Goulding M. (2015). A genetically defined asymmetry underlies the inhibitory control of flexor-extensor locomotor movements. Elife.

[21] Alvarez F.J., Jonas P.C., Sapir T., Hartley R., Berrocal M.C., Geiman E.J., Todd A.J., Goulding M. (2005). Postnatal Phenotype and Localization of Spinal Cord V1 Derived Interneurons. J. Comp. Neurol..

[22] Bikoff J.B. (2019). Interneuron Diversity and Function in the Spinal Motor System. Curr. Opin. Physiol..

[23] Gabitto M.I., Pakman A., Bikoff J.B., Abbott L., Jessell T.M., Paninski L. (2016). Bayesian Sparse Regression Analysis Documents the Diversity of Spinal Inhibitory Interneurons. Cell.

[24] Sweeney L.B., Bikoff J.B., Gabitto M.I., Brenner-Morton S., Baek M., Yang J.H., Tabak E.G., Dasen J.S., Kintner C.R., Jessell T.M. (2018). Origin and Segmental Diversity of Spinal Inhibitory Interneurons. Neuron.

[25] Benito-Gonzalez A., Alvarez F.J. (2012). Renshaw Cells and Ia Inhibitory Interneurons Are Generated at Different Times from P1 Progenitors and Differentiate Shortly After Exiting the Cell Cycle. J. Neurosci..

[26] Doe C.Q. (2017). Temporal Patterning in the Drosophila CNS. Annu. Rev. Cell Dev. Biol..

[27] Buckley D.M., Burroughs-Garcia J., Kriks S., Lewandoski M., Waters S.T. (2020). Gbx1 and Gbx2 Are Essential for Normal Pat-terning and Development of Interneurons and Motor Neurons in the Embryonic Spinal Cord. J. Dev. Biol..

[28] Boeri J., Le Corronc H., Lejeune F.-X., Le Bras B., Mouffle C., Angelim M.K.S., Mangin J.-M., Branchereau P., Legendre P., Czarnecki A. (2018). Persistent Sodium Current Drives Excitability of Immature Renshaw Cells in Early Embryonic Spinal Networks. J. Neurosci..

[29] Hoang P., Chalif J.I., Bikoff J.B., Jessell T.M., Mentis G.Z., Wichterle H. (2018). Subtype Diversification and Synaptic Specificity of Stem Cell-Derived Spinal Interneurons. Neuron.

[30] Allan D.W., Thor S. (2015). Transcriptional Selectors, Masters, and Combinatorial Codes: Regulatory Principles of Neural SubtypeSpecification. Wiley Interdiscip. Rev. Dev. Biol..

[31] Blacklaws J., Deska-Gauthier D., Jones C.T., Petracca Y.L., Liu M., Zhang H., Fawcett J.P., Glover J.C., Lanuza G.M., Zhang Y. (2015). Sim1is Required for the Migration and Axonal Projections of V3 Interneurons in the Developing Mouse Spinal Cord. Dev. Neurobiol..

[32] Zhang Y., Narayan S., Geiman E., Lanuza G., Velasquez T., Shanks B., Akay T., Dyck J., Pearson K., Gosgnach S. (2008). V3 Spinal Neurons Establish a Robust and Balanced Locomotor Rhythm During Walking. Neuron.

[33] Danner S.M., Zhang H., Shevtsova N., Borowska-Fielding J., Deska-Gauthier D., Rybak I.A., Zhang Y. (2019). Spinal V3 In-ter-neurons and Left–Right Coordination in Mammalian Locomotion. Front. Cell. Neurosci..

[34] Deska-Gauthier D., Borowska-Fielding J., Jones C.T., Zhang Y. (2020). The Temporal Neurogenesis Patterning of Spinal p3–V3 Interneurons into Divergent Subpopulation Assemblies. J. Neurosci..

[35] Müller T., Brohmann H., Pierani A., Heppenstall P.A., Lewin G., Jessell T.M., Birchmeier C. (2002). The Homeodomain Factor Lbx1 Distinguishes Two Major Programs of Neuronal Differentiation in the Dorsal Spinal Cord. Neuron.

[36] John A., Wildner H., Britsch S. (2005). The Homeodomain Transcription Factor Gbx1 Identifies a Subpopulation of Late-Born GABAergic Interneurons in the Developing Dorsal Spinal Cord. Dev. Dyn..

[37] Lai H.C., Seal R.P., Johnson J.E. (2016). Making Sense Out of Spinal Cord Somatosensory Development. Development.

[38] Buckley D.M., Burroughs-Garcia J., Lewandoski M., Waters S.T. (2013). Characterization of the Gbx1^−/−^ Mouse Mutant: A Re-quirement for Gbx1 in Normal Locomotion and Sensorimotor Circuit Development. PLoS ONE.

[39] Meziane H., Fraulob V., Riet F., Krężel W., Selloum M., Geffarth M., Acampora D., Herault Y., Simeone A., Brand M. (2013). The Homeodomain factorGbx1is Required for Locomotion and Cell Specification in the Dorsal Spinal Cord. PeerJ.

[40] Petracca Y.L., Sartoretti M.M., Di Bella D.J., Marin-Burgin A., Carcagno A.L., Schinder A.F., Lanuza G.M. (2016). The Late and Dual Origin of Cerebrospinal Fluid-Contacting Neurons in the Mouse Spinal Cord. Development.

[41] Böhm U.L., Prendergast A., Djenoune L., Figueiredo S.N., Gomez J., Stokes C., Kaiser S., Suster M., Kawakami K., Charpentier M. (2016). CSF-Contacting Neurons Regulate Locomotion by Relaying Mechanical Stimuli to Spinal Circuits. Nat. Commun..

[42] Fidelin K., Djenoune L., Stokes C., Prendergast A., Gomez J., Baradel A., del Bene F., Wyart C. (2015). State-Dependent Modu-lation of Locomotion by GABAergic Spinal Sensory Neurons. Curr. Biol..

[43] Hubbard J., Böhm U.L., Prendergast A., Tseng P.-E.B., Newman M., Stokes C., Wyart C. (2016). Intraspinal Sensory Neurons Provide Powerful Inhibition to Motor Circuits Ensuring Postural Control During Locomotion. Curr. Biol..

[44] Tripodi M., Stepien A.E., Arber S. (2011). Motor Antagonism Exposed by Spatial Segregation and Timing of Neurogenesis. Nat. Cell Biol..

[45] Knogler L.D., Ryan J., Saint-Amant L., Drapeau P. (2014). A Hybrid Electrical/Chemical Circuit in the Spinal Cord Generates a Transient Embryonic Motor Behavior. J. Neurosci..

[46] Berg E.M., Björnfors E.R., Pallucchi I., Picton L.D., El Manira A. (2018). Principles Governing Locomotion in Vertebrates: Lessons from Zebrafish. Front. Neural Circuits.

[47] Saint-Amant L. (2010). Development of Motor Rhythms in Zebrafish Embryos. Prog. Brain Res..

[48] McLean D.L., Fan J., Higashijima S.-I., Hale M.E., Fetcho J.R. (2007). A Topographic Map of Recruitment in Spinal Cord. Nat. Cell Biol..

[49] McLean D.L., A Masino M., Koh I.Y.Y., Lindquist W.B., Fetcho J.R. (2008). Continuous Shifts in the Active Set of Spinal Interneurons During Changes in Locomotor Speed. Nat. Neurosci..

[50] Roussel Y., Paradis M., Gaudreau S.F., Lindsey B.W., Bui T.V. (2020). Spatiotemporal Transition in the Role of Synaptic Inhibition to the Tail Beat Rhythm of Developing Larval Zebrafish. Eneuro.

[51] Buss R.R., Drapeau P. (2001). Synaptic Drive to Motoneurons During Fictive Swimming in the Developing Zebrafish. J. Neuro Physiol..

[52] Kimura Y., Okamura Y., Higashijima S.-I. (2006). Alx, a Zebrafish Homolog of Chx10, Marks Ipsilateral Descending Excitatory Interneurons That Participate in the Regulation of Spinal Locomotor Circuits. J. Neurosci..

[53] Kimura Y., Higashijima S.-I. (2019). Regulation of Locomotor Speed and Selection of Active Sets of Neurons by V1 Neurons. Nat. Commun..

[54] Kishore S., Fetcho J.R. (2013). Homeostatic Regulation of Dendritic Dynamics in a Motor Map in Vivo. Nat. Commun..

[55] Satou C., Kimura Y., Higashijima S.-I. (2012). Generation of Multiple Classes of V0 Neurons in Zebrafish Spinal Cord: Progenitor Heterogeneity and Temporal Control of Neuronal Diversity. J. Neurosci..

[56] Ampatzis K., Song J., Ausborn J., El Manira A. (2014). Separate Microcircuit Modules of Distinct V2a Interneurons and Motoneurons Control the Speed of Locomotion. Neuron.

[57] Ausborn J., Mahmood R., El Manira A. (2012). Decoding the Rules of Recruitment of Excitatory Interneurons in the Adult Zebrafish Locomotor Network. Proc. Natl. Acad. Sci. USA.

[58] Björnfors E.R., El Manira A. (2016). Functional Diversity of Excitatory Commissural Interneurons in Adult Zebrafish. Elife.

[59] McLean D.L., Fetcho J.R. (2009). Spinal Interneurons Differentiate Sequentially from Those Driving the Fastest Swimming Move-ments in Larval Zebrafish to Those Driving the Slowest Ones. J. Neurosci..

[60] Myers P., Eisen J., Westerfield M. (1986). Development and Axonal Outgrowth of Identified Motoneurons in the Zebrafish. J. Neurosci..

[61] Myers P.Z. (1985). Spinal Motoneurons of the Larval Zebrafish. J. Comp. Neurol..

[62] Wen H., Eckenstein K., Weihrauch V., Stigloher C., Brehm P. (2020). Primary and Secondary Motoneurons Use Different Calcium Channel Types to Control Escape and Swimming Behaviors in Zebrafish. Proc. Natl. Acad. Sci. USA.

[63] Kishore S., Cadoff E.B., Agha M.A., McLean D.L. (2020). Orderly Compartmental Mapping of Premotor Inhibition in the Developing Zebrafish Spinal Cord. Science.

[64] Pujala A., Koyama M. (2019). Chronology-Based Architecture of Descending Circuits That Underlie the Development of Locomotor Repertoire After Birth. Elife.

